# Risk factors for suicidal ideation among the older people living alone in rural region of China: A path analysis

**DOI:** 10.1097/MD.0000000000021330

**Published:** 2020-07-17

**Authors:** Caiyun Hu, Dongdong Zhao, Fengfeng Gong, Yuanyuan Zhao, Jie Li, Yehuan Sun

**Affiliations:** a Department of Scientific Research, The First Affiliated Hospital of Anhui Chinese Medical University, Heffei; b The First Affiliated Hospital of Anhui Medical University, Hefei; c Medical department, Fuyang Hospital of Anhui Medical University, Fuyang; d The Fifth Sanatorium for Retired Cadres, Anhui Provincial Military Region, Hefei; e School of Public Health, Anhui Medical University, Hefei.

**Keywords:** anxiety, depression, older people, suicidal ideation

## Abstract

In China, suicide accounts for twenty-six percent of all suicides worldwide; however, researches on the suicidal ideation among older people living alone in the rural region are few. We performed a cross-sectional study to explore the prevalence and influence factors of suicidal ideation, and provide a theoretical basis for suicide prevention among older people living alone in rural region.

695 older people living alone in rural region were selected by using stratified cluster sampling. Chi-square for categorical variables, *T*-test for continuous variables, and path analysis were conducted to statistical analysis.

The prevalence of suicidal ideation among the elderly living alone in rural China was 23.6%. Path analyses showed that depression had the most substantial influence on suicidal ideation among the elderly living alone, it was also as a mediator between physical, economic status, social support, anxiety, and suicidal ideation; anxiety and social support had both direct and indirect influence on suicidal ideation; physical status and economic status had indirection impact.

The incidence of suicidal ideation among the elderly living alone was high in Dangtu county. Psychological disorders (anxiety and depression) had the strongest impact on suicidal ideation. Strategies and measures targeting these relevant factors (economic status, physical status, and social support) should be taken to reduce the burdens of suicidal ideation among the elderly living alone in China.

## 1 Introduction

Suicide has become a serious public health and social problem worldwide, it did not only result in destroyed personal life and losing productivity but also could lead to enormous economic and health care burden on society. China is with the highest weight of suicide: the suicide rate in China in 2012 was 7.8 per 100000.^[1]^ According to the World Health Organization, in China, suicide was the fifth leading cause of death, accounted for twenty-six percent of all suicides worldwide.^[2]^ It estimated that suicide would account for 1.53 million deaths by 2020.^[3]^ Previous study indicated that the elderly were especially vulnerable to suicide.^[4]^ A meta-analysis involved 11526 subjects suggested the pooled prevalence of suicidal ideation among the Chinese elderly was 11.5% (ranging from 2.2% to 21.5%).^[5]^ With the accelerating aging of the Chinese population, there were 222 million people who were 60 and above till the end of 2015.^[6]^ Simultaneously, owing to the accelerated process of urbanization, the number of elderly parents left in rural empty nests is increasing, especially in some rural districts.^[7,8]^ In 2003, the empty nest families accounted for almost twenty-five percent of older families in China; it predicted that the proportion would reach ninety percent by 2030.^[8]^ However, older people living alone are especially disadvantaged groups of empty nest elders. Suicide mortality of rural elderly is 3 to 4 times higher than urban elderly.^[9]^ The rate of elderly suicide is highest in rural areas, which is approximately 5 times the rate in the general population.^[9]^ However, researches on the prevalence and influence factors of suicidal ideation among older people living alone in the rural region are few.

Suicidal ideation is an important evaluation index of suicidal behavior.^[10,11]^ Suicidal ideation always arises before suicide completion, and is the inevitable process of suicide. Nock et al^[12]^ found that 60% suicide plan and attempts occurred in the first year of suicidal ideation onset. Therefore, early prevention and detection of suicidal ideation are of momentous importance. Intervention in suicidal ideation may be the most effective method in preventing suicide. A lot of studies were executed to investigate probable risk factors of suicidal ideation. On socio-demographic level, annual personal income≤2200, chronic diseases, and disabled activity of daily life (ADL) status may be contributors to suicidal ideation.^[13]^ On social-environmental level, poor social support was positively associated with suicidal ideation.^[14]^ On psychosocial dimension, depression disorder has a strong connection with suicidal ideation.^[15]^ However, most of them focused on adolescent and general elderly. Few researches focused on the direct and indirect relationship between suicidal ideation and risk factors among the elderly living alone in rural China. Do these associations exist among the elderly living alone in rural area? What about the direct and indirect associations between risk factors and suicidal ideation? Accordingly, to find out these answers, the present study was conducted to provide some scientific insights, ultimately, to guide tailored treatments on suicidal ideation.

## 2 Subjects and methods

### 2.1 Subjects

A cross-sectional survey was performed in rural region of Dangtu county, located in Anhui province. The participants were selected by a cluster-sampling method, 1 of fifteen district in Dangtu was recruited, which has fourteen rural villages, details of the participants selection were introduced in our research group's previous study.^[16]^ Aged 60 years or older and living alone in that rural district for at least 1 year were included in this study. People declined to be interviewed were excluded, and with communication disorders were also not be chosen, such as hearing impairments, speech impediments. The interviewer team were given a uniform training on the investigation which comprised of fourteen undergraduate or postgraduate students. Support was acquired from the village doctors who have credibility in the local countryside, they accompanied interviewers into participants’ homes for the questionnaire. Participants were provided with an introduction of the study to obtain their informed consent. Each participant received a gift worth 10 Yuan (Chinese Yuan) to make the response rate maximize. Our study was approved by the Ethics Committee of Anhui Medical University, Hefei, China.

### 2.2 Methods

The questionnaire consisted of the following contents; after each participant completed the questionnaire, the interviewer checked on-site, and participant was asked again to complete the questionnaire if there were any omissions.

#### 2.2.1 Sociodemographic variables

Including gender, age, education level, physical status, economic status, and chronic diseases. Physical status and economic status were assessed by self-reported, with the categories as poor, general, and good.

#### 2.2.2 Suicidal ideation

Beck Scale for Suicide Ideation was used to estimate suicidal ideation.^[17]^ The beck scale for suicide ideation consists of 19-item instruments which collect the current intensity of elderly's specific attitudes, behaviors, and plans to commit suicide. Each item was answered on a 3-point scale ranging from 0 to 2, with a total score of 0 to 38. In most studies, the first 5 items were used to estimate the presence or absence of suicidal ideation.^[18,19]^ If a patient scored 0 on items 4 and 5, it means the absence of suicidal ideation; Otherwise, an individual was deemed too has suicidal ideation.

#### 2.2.3 Socio-demographic

A brief questionnaire was used to collect the participant's age, education level (illiteracy, primary, at least some middle school), living situation (alone, spouse/children, others), physical status (poor, general, good), financial situation (poor, general, good) and the number of chronic diseases (no, 1,2,>2).

#### 2.2.4 Activities of daily living

The Lawton–Brody Activities of Daily Living Scale was used to evaluate the participant's activities of daily living.^[20]^ The ADL scale contains 14-item instruments; each activity is evaluated by scoring from 1 (independent execution of activities) to 4 points (full dependence). A total score of higher than 14 was defined as disabled.

#### 2.2.5 Social support

The Social Support Rating Scale (SSRS) was applied in this study.^[21]^ The SSRS consists of 10 items, measuring 3 dimensions of social support: objective support (3 items), subjective support (4 items), and utilization degree of support (3 items). Item scores of the SSRS were simply added up; higher scores indicated better social support.

#### 2.2.6 Anxiety

Zung Self-Rating Anxiety Scale^[22]^ was used to assess the levels of anxiety in participants. There are 20 questions with each response using a 4-point scale, from “none” to “most of the time”. Scores more than 70 points mean severe anxiety, 60 to 69 points mean moderate anxiety, 50 to 59 points mean mild anxiety, and less than 40 points mean no anxiety.

#### 2.2.7 Depression

The participant's depression was measured with the Geriatric Depression Scale-15 (GDS-15).^[23]^ The GDS-15 is composed of 15 yes/no questions on functional and depressive mood symptoms, the total scores ranging from 0 to 15, GDS-15 standardized scores not less than 8 indicate significant depression.

#### 2.2.8 Quality of life

The participant's health-related quality of life was assessed by using the World Health Organization Quality of Life-abbreviated,^[24]^ it consists of 2 independent questions (assess overall quality of life and general health satisfaction) and 24 items divided into 4 subscales (physical health, psychological health, social relationships, and environment). Scores for questions range from 1 to 5; a higher score indicated better social support.

### 2.3 Data analysis

Data entry was carried out by Epidata 3.1. Statistical analyses were performed by SPSS 10.0 and AMOS 21.0. A Chi-square test was performed for categorical variables, and *T*-test was conducted for continuous variables. A path analysis was used to assess the direct and indirect association between identified risk factors and suicidal ideation. *P*-values of all statistical analyses less than .05 were considered statistically significant.

## 3 Results

### 3.1 Characteristics of participants

Among the 695 participants, suicidal ideation was defined in 164 participants (23.6%). 265 (38.1%) were male; the mean age was 72.64 ± 6.94 years (range: 60 to 94 years); 430 (61.9%) were female, the mean age was 74.51 ± 6.83 years (range: 60 to 93 years). 82.7% were illiteracy (575/695), education level of primary school and above were both 8.6% (60/695). Only 11.7% of older people reported no chronic diseases (82/695). 193 (27.8%) elder nesters were ADL disabilities. 123 (17.7%) were mild anxiety; 30 (4.3%) were at least moderate anxiety. 25% (174/695) elderly living alone reported depression. The socio-demographic characteristics of 695 participants were shown in Table 1.

**Table 1:**
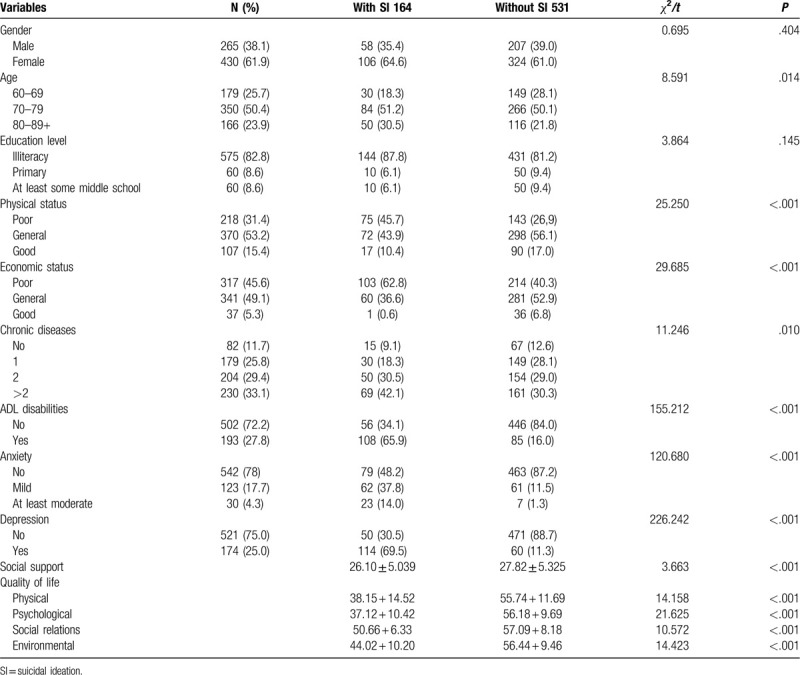
The distribution of the suicidal ideation among the older people alone of different demographic characteristics (%).

### 3.2 Potential correlative factors in association with suicidal ideation

Age (*χ*^*2*^ = 8.591, *P* = .014), physical status (*χ*^*2*^ = 25.250, *P* < .001), economic status (*χ*^*2*^ = 29.685, *P* < .001), number of chronic diseases (*χ*^*2*^ = 11.246, *P* = .010), ADL disabilities (*χ*^*2*^ = 115.212, *P* < .001), anxiety (*χ*^*2*^ = 120.680, *P* < .001) and depression (*χ*^*2*^ = 226.242, *P* < .001) were risk factors for suicidal ideation. Social support (*t* = 3.663, *P* < .001) and quality of life (physical aspect: *t* = 14.158, *P* < .001; psychological aspect: *t* = 21.625, *P* < .001; social relations: *t* = 10.572, *P* < .001; environmental aspect: *t* = 14.423, *P* < .001) were protection factors among older people living alone. However, gender (*χ*^*2*^ = 0.695, *P* = .404) and education level (*χ*^*2*^ = 3.864, *P* = .145) were not significantly associated with suicidal ideation.

Detailed information was summarized in Table 1.

### 3.3 Factors associated with suicidal ideation: a path analysis

**Figure 1:**
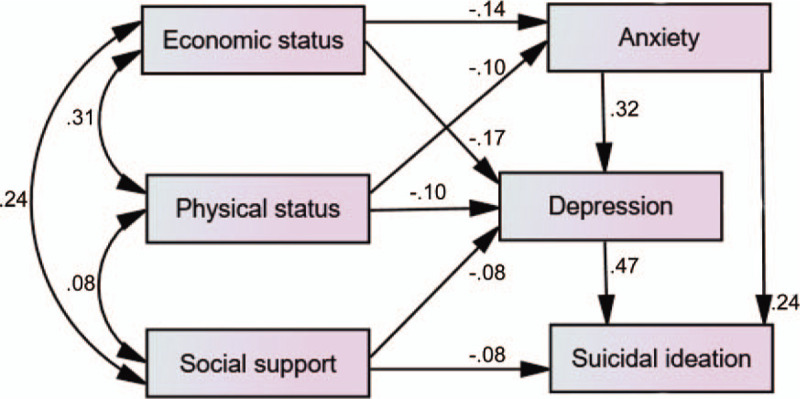
Factors associated with suicidal ideation by path analysis. Standardized effects.

A path analysis was conducted for comprehensively understanding the associations between risk factors and suicidal ideation in the elderly living alone. This model included 5 factors (Fig. 1.): depression, anxiety, social support, economic status, physical status. The model suggested that anxiety and social support had both a direct and indirect impact on suicidal ideation. Economic status and physical status only had indirect influence mediated by anxiety and depression. Depression imposed a direct influence on suicidal ideation in the elderly living alone. Five variables’ effect on suicidal ideation was in Table 2. These variables combined path represented an acceptable overall fit for the data (*χ*^*2*^ = 2.160, *df* = 3, *P* = .540, *CFI* = 1.00, *TLI* = 1.00, *RMSEA*<0.001), explained 38.3% of the variance (*r*^*2*^ = 0.384). The final path model adequately represented the data.

**Table 2:**
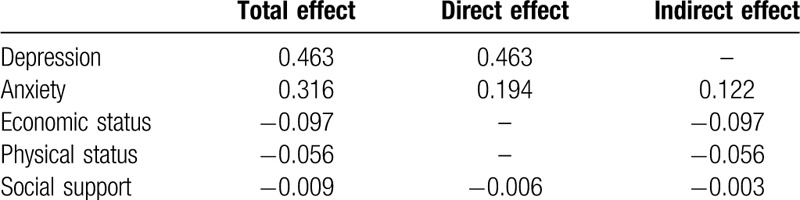
Standardized effects on suicidal ideation from path analysis among the older people living alone in rural China.

## 4 Discussion

This study investigated 695 elderly living alone in rural areas of Anhui province to explore the prevalence and potential relevant factors of suicidal ideation. As far as we know, this is the first study focusing on rural older people living alone in China. The prevalence of suicidal ideation was 23.6% among older people living alone, much higher than previous studies. Dong et al^[5]^ conducted a meta-analysis on suicidal ideation of the Chinese elderly and reported that the prevalence was 14.7% among general elderly living in rural areas; Another meta-analysis focused on the prevalence of mental health problems among Chinese medical students reported that the suicidal ideation prevalence of medical students was 11%.^[25]^ It was also higher than Noguchi et al^[15]^ and Lemstra et al,^[26]^ who reported the prevalence of suicidal ideation among Japanese rural elderly was 10.0%, and Canadian rural elderly was 11.9%, respectively. In domestic, several reports about the prevalence of suicidal ideation among seniors were also lower than ours: 2.3% in Beijing,^[27]^ 5.5% in Hong Kong,^[28]^ and 9.4% in US Chinese populations.^[29]^ The extremely high incidence in our study implied that suicidal ideation was a serious problem, especially among the elderly alone in rural region in China.

The results of this study indicated that ADL disorders had a significant positive association with suicidal ideation among older people living alone. It's in accordance with Awata's study.^[30]^ Impaired ADL increased the psychological and physical pain among older people living alone, also the burden of their children; therefore, those adverse consequences of impaired ADL may exacerbate the stress contributing to suicidal ideation. Additionally, we found that age was significantly correlated with suicidal ideation, Dong et al^[29]^ had the same conclusion. The age-related cognitive and physical decline may increase their dependence on children or someone else; however, the elderly living alone can’t get enough care from others. Local government's timely visit and help to the elderly living alone are of great importance. More frequent home visits by off-springs and commissioned welfare volunteers is not a bad choice, too.^[14,31]^ A previous study mentioned that the presence of arthritis and renal failure was significantly associated with a higher risk of suicidal ideation.^[32]^ From another perspective, our study showed that the number of chronic diseases was associated with suicidal ideation. Chronic diseases adversely affect the quality of life.^[33]^ This study also revealed that 4 fields of life quality were significantly associated with suicidal ideation among older people living alone in the rural region. A possible explanation may be as follow: quality of life at a good level makes the elderly full of enthusiasm and hope; thus, the incidence of suicidal ideation will reduce.

This path analysis found that economic status had an indirect impact on suicidal ideation. Researches of Li^[34]^ and Hunng et al^[35]^ indicated those with good economic status had decreased suicidal ideation. Chen's study got a parallel conclusion: the elderly with poor economic status were liable to suicidal ideation.^[36]^ A path analysis conducted by Ge et al^[15]^ confirmed our result, too: depression was found to play a mediating role between the economic difficulties and suicidal ideation. The implication of this finding was that some poverty-relief policies and measures were of essential importance in preventing the appearance of suicidal ideation. Similarly, health status was inversely correlated with suicidal ideation in an indirect way. The article targeting the US Chinese elderly people acquired the same result: lower heath status was positively correlated with suicidal ideation.^[29]^ When poor physical status interacts with poor economic status, the aging people give themselves up as hopeless; hence, cheaper medicine fees and guidance for daily physical training from the local department are needed. As to social support, the previous study implied that seniors with poor social support might be mediated by depressive symptoms, then had an indirect influence on suicidal ideation.^[37]^ A direct association was also found in our study. It may be a reflection that social welfare support is getting more and more indispensable.

In our path analysis, economic status and physical status imposed indirect influence on suicidal ideation, which was mediated by anxiety and depression. While social support was only mediated by depression. Overall, depression was the most important risk factors of suicidal ideation among the older people living alone in rural China, which was followed by anxiety. Psychological suffering might be the most unbearable factor in the olderl group alone in a rural region. Borges study in Mexico found mental disorder (anxiety and depression) was a common factor associated with both lifetime and 2-week prevalence of suicidal ideation.^[38]^ Ibrahim study among adolescents in Malaysia revealed the role of psychological factors in predicting suicidal ideation.^[39]^ All the above proved the stability of the association across culture and age. As anxiety and depression played crucial roles, corresponding early prevention, intervention, detection, and treatment are vital in preventing suicide, especially for those with poor physical and economic status.

Several limitations of this study need to be acknowledged. First, a cross-sectional designed study had limited power to determine the cause and effect between identified factors and suicidal ideation. Second, the present study was just conducted in Anhui Province of China, the generalizability of the study results is limited; the further research with larger population conducted into the broad areas in China is warranted. Third, several factors, such as economic status and chronic disease were self-reported. Report bias may influence the stability of outcomes.

It could not be denied that results of this study have important public health policy implications, the findings identify possible targets to reduce the risk of suicidal ideation through the treatment of psychiatric disorders such as depression and anxiety, and improving the life quality of older people living alone. Besides, governments should accelerate the establishment of a sound social security system, strengthening care and increasing financial input for the elderly living alone in rural regions, concerned about their physical and mental health, and actively carry out mental health education in rural regions to reduce the incidence of suicidal ideation in rural older people living alone.

## Acknowledgments

We would like to thank the elderly who participated in this cross-sectional study. We also thank all the village doctors for their generous assistance with data collection process.

## Author contributions

**Conceptualization:** Caiyun Hu.

**Formal analysis:** Dongdong Zhao.

**Investigation:** Caiyun Hu, Fengfeng Gong, Jie Li.

**Methodology:** Yuanyuan Zhao.

**Project administration:** Yuanyuan Zhao, Jie Li.

**Supervision:** YeHuan Sun.

**Writing – original draft:** Caiyun Hu, Dongdong Zhao.

**Writing – review & editing:** YeHuan Sun.
